# COVID-19 and corticosteroids: a narrative review

**DOI:** 10.1007/s10787-022-00987-z

**Published:** 2022-05-13

**Authors:** Gaber El-Saber Batiha, Ali I. Al-Gareeb, Hebatallah M. Saad, Hayder M. Al-kuraishy

**Affiliations:** 1 Department of Pharmacology and Therapeutics, Faculty of Veterinary Medicine, Damanhour University, Damanhour, 22511 AlBeheira Egypt; 2 Department of Clinical Pharmacology and Medicine, College of Medicine, Mustansiriyiah University, Baghdad, Iraq; 3 Department of Pathology, Faculty of Veterinary Medicine, Matrouh University, Matrouh, 51744 Matrouh Egypt

**Keywords:** Corticosteroids, COVID-19, Comorbidities

## Abstract

It has been reported that corticosteroid therapy was effective in the management of severe acute respiratory syndrome (SARS) and the Middle East Respiratory Syndrome (MERS), and recently in coronavirus disease 2019 (COVID-19). Corticosteroids are potent anti-inflammatory drugs that mitigate the risk of acute respiratory distress syndrome (ARDS) in COVID-19 and other viral pneumonia, despite a reduction of viral clearance; corticosteroids inhibit the development of cytokine storm and multi-organ damage. The risk–benefit ratio should be assessed for critical COVID-19 patients. In conclusion, corticosteroid therapy is an effective way in the management of COVID-19, it reduces the risk of complications primarily acute lung injury and the development of ARDS. Besides, corticosteroid therapy mainly dexamethasone and methylprednisolone are effective in reducing the severity of COVID-19 and associated comorbidities such as chronic obstructive pulmonary diseases (COPD), rheumatoid arthritis, and inflammatory bowel disease (IBD).

## Background

Early studies revealed the use of glucocorticoids in the treatment of severe acute respiratory syndrome (SARS) and acute respiratory distress syndrome (ARDS) lead to comparable results. Glucocorticoid therapy at a high dose was followed in managing the SARS epidemic in China (Meng et al. 2003).

Several studies recommended glucocorticoid curative doses in the period of the SARS eruption that were far higher fairly accurate the healing approved dosages in the management of asthma and respiratory failure (Meng et al. 2003). Following a single comparative report, high-dose glucocorticoid treatment was shown to be far more effective than low-dose treatment (Xiao et al. 2004). It was established that systemic glucocorticoid therapy in the management of SARS may be associated with injury (Xiao et al. 2004). While in a comparative study, hydrocortisone therapy, when started early, was related to elevated consequent bacterial infections (Hart et al. 2011). In the meantime, prolonged high-dose glucocorticoid treatment led to immunosuppressive effects, with the subsequent probable appearance of severe microbial infections among others (König et al 2021). Besides the complications after glucocorticoid therapy, it was administered to seriously patients with Middle East Respiratory Syndrome (MERS) to overcome respiratory failure and avoid lung injury. Nevertheless, no study provided evidence of the success of this approach (Meduri et al. 2009; Dube et al. 2020). Unfortunately, glucocorticoid treatment was reported to increase mortality and retard RNA viral elimination in patients with Middle East Respiratory Syndrome-Coronavirus (MERS-CoV) (Al-Tawfiq and Memish 2017). A noteworthy number of patients exposed to 2019-CoV developed serious respiratory distress and necessitated rapid intervention. But the management of SARS with glucocorticoid remains divisive due to contradictory findings. In addition, its efficacy in the treatment of acute lung damage remains unclear so far. Conversely, a systematic review reported that long-term glucocorticoid therapy leads to positive results (Yang et al. 2020a, b). In different studies, glucocorticoid has been evidenced to reduce ARDS-associated inflammation, and significantly improve lung and extrapulmonary organ dysfunction, also reduce oxygen need and hospitalization period (Meduri et al. 2016). Methylprednisolone therapy could increase positive modulation of systemic inflammation by inducing improvement (Meduri et al. 2002). Recently, an in vivo study revealed that glucocorticoids decreased inflammation and reduced acute lung injury, as well as mortalities throughout the early stage of the serious illness (Marik et al. 2011). Nevertheless, based on previous reports, glucocorticoid treatment in ARDS is not required and could exacerbate the clinical feature. Hydrocortisone has been shown to improve lung performance following septicemia-related respiratory failure, although no remarkable benefits have been noted in these patients (Marks et al. 2003). Unfortunately, it was reported that glucocorticoids resulted in a significant mortality rate in ARDS patients compared to those not receiving high-dose glucocorticoids. However, the efficacy of glucocorticoids has not been investigated in the same categories of patients, as well as the same stages of the illness conditions by the existing studies on ARDS (Elbrassi et al. 2020). Concurrent use of glucocorticoids in precise illness following ARDS has reduced the number of deaths (Gurganus et al. 2019). Moreover, it is necessary to monitor administered glucocorticoid doses, since elevated-dose treatment could raise oxygen reliance, and probably lead to worse results (Harrington 2020). During the SARS outbreak in 2003, patients treated with high-dose glucocorticoids developed osteonecrosis closely related to the doses they received (Chan et al. 2006). Furthermore, neuropsychiatric conditions have been reported following high-dose treatment (Kothgassner et al. 2021). Interestingly, several studies indicated that treatment at appropriate doses defined by the severity of the disease could improve outcomes when lung damage was effectively managed (Mokra et al. 2019).

## Pharmacology of corticosteroids

**Fig. 1:**
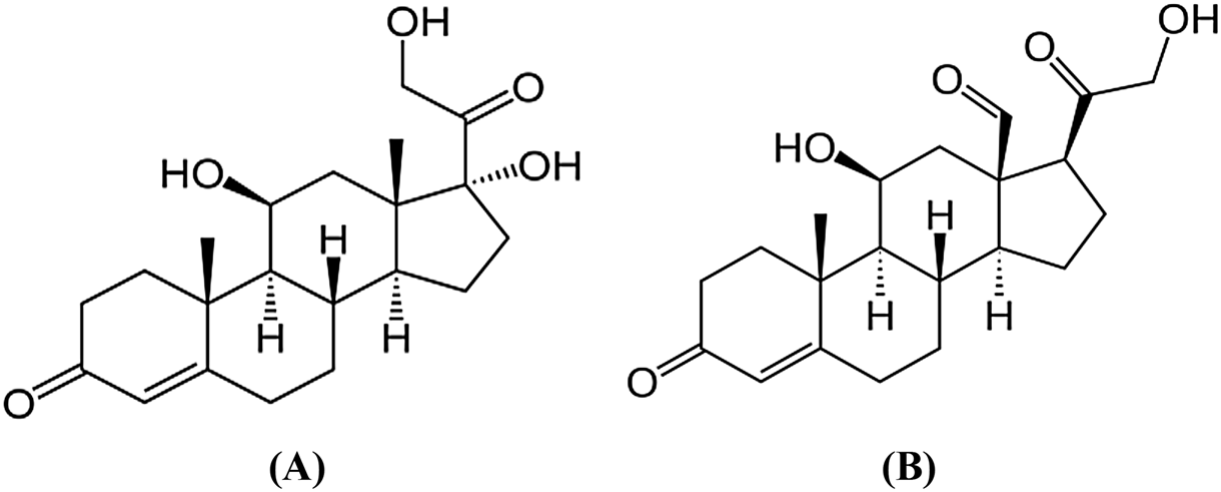
Chemical structures of main corticosteroids: **A**: cortisol, **B**: aldosterone

Corticosteroids are a class of steroid hormones produced from adrenal cortex. The chief classes of corticosteroids are mineralocorticoids and glucocorticoids which involved in a wide variety of physiological functions including immune response, regulation of inflammation, body metabolism, electrolyte balance, stress, and behavior (Whitehouse 2011; Williams 2018). Naturally occurring corticosteroids including cortisol, corticosterone, cortisone and aldosterone, though the main corticosteroids produced from adrenal cortex are cortisol and aldosterone (Djaballah-Ider and Touil-Boukoffa 2020) (Fig. 1).

There are two main classes of corticosteroids which are glucocorticoids like cortisol and mineralocorticoid like aldosterone (Williams 2018). Of note, cortisol possesses anti-inflammatory, antiproliferative, vasoconstrictor, and immunosuppressive effects with noteworthy catabolic effects. The anti-inflammatory effect of cortisol is mediated by induction release of anti-inflammatory mediators (transactivation) and blocking release of pro-inflammatory/inflammatory mediators (trans-repression) (Whitehouse 2011). The immunosuppressive effect of cortisol is mediated by direct suppression of T cell function with inhibition of delayed hypersensitivity reactions. Though, the antiproliferative of cortisol is mainly mediated by inhibition of epidermal cell turnover and DNA synthesis. The vasoconstrictor effect of cortisol is done by suppression release of histamine. Besides, aldosterone regulates ion and electrolyte transports across renal epithelial cells (Whitehouse 2011).

**Fig. 2:**
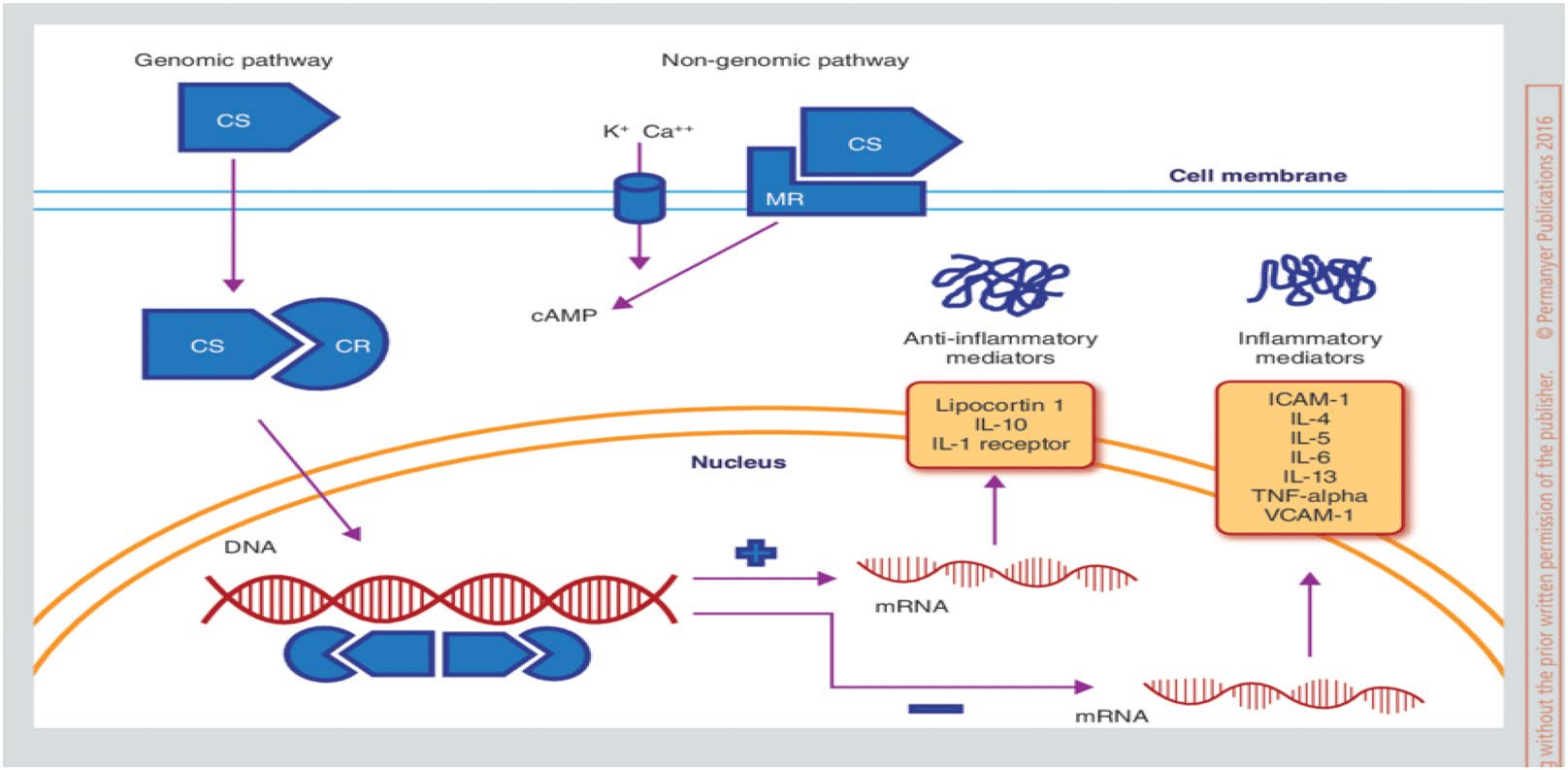
Mechanism of corticosteroids action: corticosteroid (CS) activates corticosteroid receptor (CR) with activation expression of DNA; this effect activates anti-inflammatory mediators and inhibits inflammatory mediators (Sibila et al. 2015)

The mechanism of corticosteroids action is though activation of glucocorticoid and mineralocorticoid receptors by cortisol and aldosterone, respectively (Williams 2018). In addition, corticosteroids may possess progestogenic activity producing sex-related adverse effects (Whitehouse 2011) (Fig. 2).

Corticosteroids are indicated in the management of different pathological conditions including:

- Allergic diseases: asthma, contact dermatitis, angioedema, allergic rhinitis, and hypersensitivity pneumonitis (Filaretova et al. 2006).
- Endocrine diseases: Addison disease, acute adrenal insufficiency, and congenital adrenal hyperplasia (Bondy 2020)
- Gastrointestinal disorders: ulcerative colitis, Crohn disease, and autoimmune hepatitis (Filaretova et al. 2005).
- Hematological diseases: lymphoma, leukemia, autoimmune hemolytic anemia and idiopathic thrombocytopenia (Malpica and Moll 2020).
- Rheumatological disorders: rheumatoid arthritis, systemic lupus erythematosus, polymyositis, vasculitis and polymyalgia rheumatica (Fokam et al. 2021).
- Ophthalmological disorders: optic neuritis, uveitis, and keratoconjunctivitis (Scherer et al. 2014).
- Miscellaneous disorders: multiple sclerosis, nephrotic syndrome, chronic hepatitis, cerebral edema and lichen planus (Shah et al. 2009).

Moreover, prolong use of corticosteroids is associated with development of adverse effects including osteoporosis, osteonecrosis, hypertension, Cushing syndrome, glaucoma, cataract, secondary diabetes mellitus, immune deficiency and secondary bacterial infections (Williams 2018).

## The link between immune-suppressive and stimulating drugs in COVID-19

The probable unhelpful property of corticosteroids, in the management of COVID-19, remains divisive since the beginning of the epidemic. Corticosteroids play a key role in immunity and inflammation, especially at low doses. During the epidemic, corticosteroid therapy has been extensively tested because it has been reported to regulate a multiplicity of cytokines involved (Brattsand and Linden 1996). Several in vivo studies reported a remarkable reduction in immunopathological lesions, although the crucial problem was the progression of viral rebound and associated adverse effects (Banuelos et al. 2017). A recent in vitro dexamethasone experiment has recommended that early corticosteroid administration can effectively improve acute inflammatory response; however, long-term treatment may enhance viral replication (Frank et al. 2010). Meanwhile, an independent Chinese study showed that early treatment with high-dose corticosteroids and quinolone had the best results for patients (van der Linden et al. 2003). However, a review reported that corticosteroids have not been suggested in the treatment of other viral illnesses as well as Dengue reported that the glucocorticoid-mediated stimulation of the hypothalamic–pituitary–adrenal axis may lead to lymphocytopenia or aggravate pro-inflammatory responses which ultimately provoked a critical outcome (Shang et al. 2020).

Early corticosteroid therapy was experienced in 132 out of 213 qualified subjects. A noteworthy decrease in average hospitalization duration was also observed in the treatment cluster. The above therapy outcome was perceptible within every individual part of the composite endpoint. It was concluded that an early acute methylprednisolone therapy towards COVID-19 diminished intensification of concern and enhanced results (Wang et al. 2020a, b).

Corticosteroid efficacy was investigated and its reasonable administration to patients with COVID-19 was confirmed (Chroboczek et al. 2021). The records of COVID-19 patients with specific findings were assessed for their features, course of therapy, and their relation with the findings. Almost 95 out of 649 patients had passed away. The risk of progressive mortality and further apparent irregularities in survival markers were reported in older male patients (Ranjbar et al. 2021). A study comparing corticosteroid treatment among dissimilar groups found that non-living individuals received a high dose compared to surviving patients (Fujishima 2020). The previous study reported intensive common use of corticosteroids in non-living patients while surviving patients treated with corticosteroids had long-term hospitalizations (Forrest et al. 2019). The mid-point time extent for temperature re-establishes for non-survivors following corticosteroid therapy was documented to be far longer than that of both survivors. In patients treated with corticosteroids, the number of lymphocytes at the entrance was lower than patients who did not receive it (Wu et al. 2020). Interestingly, the immune response has improved considerably following the treatment of patients who survive, contrary to all expectations in non-survivors. It can be concluded that COVID-19 patients with different results responded to corticosteroids in a variety of ways. Corticosteroids were administered preferably to the survivors, while lower lymphocytic count and reduced corticosteroids effect were recorded in non-survivors (Hui et al. 2019). Corticosteroid therapy in surviving patients with prolonged hospitalization period, the lymphocytes have improved (Rubio-Rivas et al. 2020). Therefore, the improved immune system, as well as temperature following corticosteroid therapy, could predict the forecast of COVID-19 patients (Al-kuraishy et al. 2020a). It was recorded that dead was frequently recorded in older male patients with elevated impacts of several persistent risk factors. Patients who did not survive had an average age of 68, noteworthy higher than that observed in surviving patients. Interestingly, a study about the causes of COVID-19 death, with a less important number of patients, reported the key role of old age and comorbidities in COVID-19 related death (Shim et al. 2020; Al-kuraishy et al. 2020b). Unfortunately, the obvious immunodeficient system in aged patients as well as impaired functioning of organs following primary illnesses has been shown to increase their susceptibility to disease evolution and insensitivity to existing therapy (Singanayagam et al. 2018). Several experimental studies reported an improved neutrophil count and reduced lymphocyte count in non-surviving COVID-19 patients (Zhang et al. 2021). Moreover, patients with SARS and MERS had lymphocytopenia, since their lymphocytes were reported to be destroyed by SARS-CoV and MERS-CoV (Liang et al. 2020). A report study showed that it was more frequently administered in non-surviving patients and serious/crucial-type surviving patients compared with the general-type surviving patient's group (Pedersen and Ho 2020). The use of corticosteroids to treat viral pneumonia, and respiratory syndromes is still divisive; it was shown to retard viral RNA elimination as in COVID-19 as respiratory syndromes (Li et al. 2020).

## Effectiveness of corticosteroid therapy in COVID-19

An analytical survey was carried out in Chinese specialized units between January and February 2020 found 11 out of 31 patients were administered corticosteroid therapy, a ratio comparable to that documented in a previous report (Wu et al. 2020). Notably, 70% of patients with COVID-19 in crucial conditions were commonly administered corticosteroids therapy (Wu et al. 2020). Unfortunately, corticosteroid therapy has been shown to be as risky as evidenced by an increase in clinical features, an elevated inflammation index, and increase irregularities on chest CT (Hu et al. 2020a, b, c). Therefore, its use was associated with the observed severe symptoms. A different report has confirmed this practice, since 33% of patients with symptoms of COVID-19 longer than 10 days were administered corticosteroid, at the time just 17% of those who had symptoms for less than 10 days (Zha et al. 2020). A recent report has evidenced that corticosteroids had no effect on virus elimination time, hospitalization period, or extent of symptoms in moderate cases (Coyle et al. 2020). ARDS has been shown to be the main cause of deadly COVID-19 cases according to pathology results (Al-kuraishy et al. 2021c). Fortunately, premature corticosteroids therapy could be associated with a decrease in the threat of ARDS in virus diseases (Wang et al. 2020a, b). Nevertheless, the effectiveness of premature corticosteroid treatment in ARDS prevention could not be confirmed, given that it was not detected in any patient (Sheianov et al. 2020). It has been reported that virus elimination extent was increased in patients with persistent hepatitis B virus (Karimi-Sari and Rezaee-Zavareh 2020). Considering the reduced size of patients’ infections (two), it was not possible to draw concrete findings. However, a previous study has reported an identical relationship for patients with SARS-CoV (Hu et al. 2020a, b, c).

The intensive use of corticosteroids in preventing pulmonary damage following severe pneumonia was because of the outstanding efficiency shown in the inhibition of excited and dysfunctional systemic inflammation as confirmed by reduced all-cause death, the impact of septic shock, and a necessity for mechanical aeration with no escalating menace of undesirable effects (Hu et al. 2020a, b, c). Previous reports recommended low dose, long-term administration as an ideal treatment towards severe pneumonia (So et al. 2020). The effectiveness of corticosteroids therapy for COVID-19 was not confirmed by a number of scholars given that observational studies and systematic reviews have not evidenced their conclusive findings again viral pneumonia (such as SARS, MERS, and H1N1) (Bauer 2005). Moreover, recent studies reported that pulse-dose treatment or prolonged use of a high dose of glucocorticoids in the premature phase may be dangerous (Herold et al. 2006; Al-kuraishy et al. 2021d). Unfortunately, these findings unnoticed the successful effects of corticosteroids on various patients, in particular those presenting serious symptoms, since it has been shown that the clinical outcomes were associated with severities of diseases, the extent of assistance, the dosage, and length of corticosteroid treatment (Meduri et al. 2018). One should keep in mind that low-dose corticosteroid therapy failed to decrease death caused by septic shock due to early lung diseases, though might take advantages to upcoming results, including premature setback of shock, decrease the time of way out from intensive care unit (ICU) and automatic aeration (Polat et al. 2017). In addition, rescue corticosteroids therapy for seriously ill patients with highly developed ARDS may improve pulmonary fibrosis and avoid increasing pathological decline (Richeldi et al. 2003), therefore, providing strong skills to support why rescue corticosteroids therapy improves conditions of several crucial patients with SARS disease. Very significant, death advantage favored the serious HIN1-disease in the therapy group which received a low dose of corticosteroids (Wang et al. 2014). Obviously, these findings robustly recommended that appropriate administration of the low dose of corticosteroids improved the conditions of crucial patients with COVID-19. Nevertheless, the above therapy may rigorously be applied in the management of COVID-19 with obvious signs (including obstinate ARDS, sepsis, or septic shock) as prescribed by suggested guidelines.

The likely efficiency of corticosteroids has recently been investigated in the management of critically ill patients having COVID-19 (Yang and Lipes 2020). The above-mentioned patients were immediately treated with corticosteroids following the admittance of COVID-19 patients. But the report revealed that the number of deaths recorded was not far different from that recorded after MERS cases not receiving the same treatment, signifying that corticosteroids treatment is not effective in ICU death in crucial COVID-19 patients (Gangopadhyay et al. 2020). However, in the meantime, regular corticosteroids treatment might increase oxygen supply. Unfortunately, this treatment was not reported to provide vital benefits to critical COVID-19 patients (Mattos-Silva et al. 2020). However, corticosteroids therapy in patients with ARDS was shown to improve furious inflammatory storm and reduce the length of management of disease and avoidance of further multi-organ injury and shock, suggesting that corticosteroids exhibited a synergistic effect in combination with other intensivists’ therapy against serious and critical COVID-19 patients (Sterne et al. 2020).

Any precise medical directive supported corticosteroids in seriously ill patients. However, subjective know-how from the previous outbreak’s treatment confirmed the accurate use of corticosteroids in the treatment of COVID-19 (Prescott and Rice 2020). Adapted medication policy should include, and not be restricted to, precise recommendations, timing, and extent, as well as remedial scrutinizing of corticosteroids treatment. Therefore, corticosteroids are not advised without guidelines for mild or serious ARDS, sepsis or septic shock, partly in accordance with clinical guidelines from World Health Organization (WHO) (Villar et al. 2020). In addition, corticosteroids treatment is not recommended for mild and early-stage ARDS since it has been shown that premature corticosteroids therapy might retard the elimination of virus and raise death threat, and corticosteroids were confirmed to exhibit probably high effect on inflammatory conditions causing lung damage as well as intestinal fibrosis at tardy-phase of the disease (Hoyles et al. 2006). Additionally, corticosteroids treatment has been shown to be related to side effects in a dose-dependent manner, especially at higher or repeated extreme doses (Manson et al. 2009). A recent study reported that minor dose and brief extent associated with checking of undesirable medicine effect provided a significant advantage in the treatment at worse-stage COVID-19 (Vounotrypidis 2020). Furthermore, a continuing record (6 months to 3 years) was proved to be necessary to recognize late side effects in patients with COVID-19.

## Corticosteroid therapy in COVID-19 with ARDS

There is clear evidence that deregulated inflammatory conditions along with coagulation associated with COVID-19 are comparable with that of ARDS, while the capacity of corticosteroid treatment (CST) in decreasing inflammation–coagulation–fibroproliferation and increasing illness improvement has been confirmed (Villar et al. 2020). Furthermore, corticosteroids therapy has been shown to exhibit positive outcomes towards inflammation-related lung illness (Meduri et al. 2009). It has been reported that COVID-19 induced an increase in cytokine level evoking less important hemophagocytic lymphohistiocytosis, a situation reactive to CST (Wohlfarth et al. 2019; Al-kuraishy et al. 2021e). Unfortunately, no previous finding has provided comprehensive support on the WHO declaration which did not suggest regular corticosteroids therapy in viral pneumonia conditions outside of clinical experiments (Yang et al. 2020a, b). Notwithstanding the existing literature supporting corticosteroids therapy in non-viral ARDS, the WHO is not in favor of CST for COVID-19-related ARDS (Hu et al. 2020a, b, c). The confirmed methodology used by WHO to take an unconditional resolution with probably severe public consequences presented several limitations. In fact, an observational experiment has demonstrated a possible reduction of viral elimination, providing a short argument to the WHO’s prescription and questionable results from a backward-looking empirical experiment with no prior established experimental procedure and subdued to confounding (Yousefifard et al. 2020). According to a current high-class investigation, a significant relationship was established between CST and likely confounders for considered results, as well as sickness brutality and comorbid diseases (Liao et al. 2016). Hence, confounding by suggestion was expected to be a noteworthy prejudice in experiments that merely afforded unfamiliar outcome estimates. Furthermore, duration of hospitalization, antiviral administration, the occurrence of respiratory collapse preceding corticosteroids treatment, and the justification for corticosteroids therapy or course of therapy were lightly recorded by previous findings (Kudo et al. 2012). It has been reported that corticosteroids therapy in patients with influenza-amplified death and nosocomial infection, which is mostly associated with elevated corticosteroids doses [generally > 40 mg methylprednisolone (or equivalent) per day] (Kudo et al. 2012). Inflammatory dysfunction was regarded as causing mortality following COVID-19. However, no report has recorded any relationship between late viral elimination and aggravated ending in crucially sick patients, while none of the available data has proved its significant harmful effect when compared with the host personal “cytokine storm” (Narain et al. 2020). A convincing record on corticosteroids therapy in current serious viral epidemics, coauthored by a member of the WHO panel on experimental management for COVID-19 reported no positive effect of corticosteroids therapy in infected persons (Tang et al. 2020). However, this explanation is subjective and with no evidence. Primarily, they concluded on 4 out of 29 studies, meanwhile, 6 out of 10 reports provided no explanatory support (Singh et al. 2020). Second, their conclusive findings did not consider the encouraging outcomes on SARS and influenza H1N1 pneumonia which recorded an interesting decrease in death in accordance with previous findings (Brun-Buisson et al. 2011). A previous study including SARS reported secure CST with reduced risk of mortality by 47% following modification of likely confounders (Auyeung et al. 2005). An experiment was performed about H1N1, in which 1055 patients were administered corticosteroids, while 1,086 were not. It has been reported that CST at lower or moderate doses remarkably decreased death (Quispe-Laime et al. 2010).

A small number of current reports based on observation have untimely recorded evidence on COVID-19 ARDS. It was recently demonstrated that COVID-19 patients with ARDS reported decreased menace of mortality following methylprednisolone therapy (doses comparable to the protocol previously suggested (Prescott and Rice 2020; Al-kuraishy et al. 2021f). Moreover, based on the records from intensivists in forefront of the pandemic in China, afford convincing evidence of the positive impact of corticosteroids therapy towards COVID-19 pneumonia validated by experts (So et al. 2020). Ultimately, methylprednisolone/dexamethasone was approved in the treatment of COVID-19 ARDS (Mongardon et al. 2021). In the end, humanity is experiencing a decisive moment with rapid ICU saturation, even in developed countries, where intensivists are forced to make exceptional decisions out of conflict areas. Corticosteroid therapy was recommended in the management of COVID-19 even though there is a discrepancy in the current literature. Nevertheless, the lack of satisfactory proof did not discredit its possible positive effect in ARDS after COVID-19 (Chaudhuri et al. 2021). Therefore, one should take into consideration the proof of non-viral origin of ARDS, the previous Chinese outcomes, as well as suggestions from Chinese and Italian physicians on forefronts. Even if strong evidence is not available, CST should still be considered in the management of COVID-19 ARDS.

Corticosteroids therapy practices recommended methylprednisolone and dexamethasone (Hasan et al. 2020a, b). In fact, methylprednisolone was administered IV, but from day 5, it was possible to give it per os. However, it was noted that the drug absorption could be compromised if given per os, following extubation. In such a case, it was necessary to control inflammation, ARDS, and impairment of organs functioning (Halpin et al. 2020). There was a vital need for the reuse of corticosteroids following a slight decrease. A current treatment approach recommended dexamethasone for no more than 10 days. However, it is not recommended to decrease doses to reduce the threat of reappearance of inflammatory conditions (Ye et al. 2020).

## Doses of corticosteroids in COVID-19

Herein, clinical data of 78 COVID-19 patients hospitalized were recorded. Among 78 patients, 55 were confirmed with general COVID-19 and the remaining 23 presented severe COVID-19. The standard therapy was administered to all the patients. Besides, patients with serious COVID-19 received the required helpful therapy. A subset of patients was treated with corticosteroids, depending on the severity and the personal appreciation of the clinicians. Further serious patients received corticosteroids, provoking contradictory features (risk factors and experimental outcomes) between them and those who were following different treatments. Critically ill patients were given corticosteroids orally; meanwhile, the others were treated with the upper dose for the long term, given that they were admitted to the hospital at the beginning of the outbreak and tended by the same clinicians. Viral elimination was evaluated using reverse transcription-polymerase chain reaction (RT-PCR). The test was repeated to prevent false-negative outcomes when negative results were obtained (Villar et al. 2020). At the moment, an independent comparative study revealed no major discrepancy following treatment with corticosteroids or not. The likely long-term implication of corticosteroids treatment in prolonging viral elimination during lung inflammation is still divisive (Yang et al. 2020a, b). The above event was previously mentioned in respiratory syndromes, though; the outcomes were dose-dependent (Rodríguez-Baño et al. 2021). Interestingly, additionally, an observational experiment reported that low-dose corticosteroid was associated with worsening (Cheng et al. 2020a, b). Corticosteroid therapy has been shown to retard viral elimination in comparison with control. This report showed better outcomes contrary to those recorded in the management of MERS (Ma et al. 2020). Then, both the above studies reported on patients with various cruelties of diseases; meanwhile, further investigations are required to verify the better results of corticosteroid toward worse cases. In addition, no protocol was conceived to manage MERS. All patients included in this study were cared in accordance with the approved protocol to pass up errors. Unfortunately, the above retrospective studies inevitably had some restrictions. Overall, the implication of low-dose corticosteroids in retarding microbial elimination during COVID-19 remained unclear. Nevertheless, it was necessary to clarify the above statement by conceiving a clear study including a great number of participants by means of long-term follow-up (Zhou et al. 2020).

It has been shown that reduction in dosage of glucocorticoids treatment could modify viral clearance duration in lung inflammation caused by COVID-19 via anti-inflammatory effect (Kumar et al. 2020; Al-kuraishy et al. 2021d). Besides, it has been demonstrated a decrease in mortality threat caused by COVID-19 pneumonia and ARDS following methylprednisolone therapy (Veronese et al. 2020). Notably, it was certified that low-dose corticosteroids therapy has likely positive effects on critical cases of COVID-19 pneumonia (Veronese et al. 2020). Even though this is a vital concern considering the complexity of managing cases, it is imperative to moderate methylprednisolone administration given that many questions remained unanswered. Hence, to tackle this matter a comparative backward trial was carried out. As a result, any noteworthy improvement was perceptible following treatment with methylprednisolone. Furthermore, oxygen supply decreased considerably in the presence of methylprednisolone treatment, which also induced a quicker reduction of c reactive protein and interleukin-6 (IL-6), though no noteworthy variation exists when compared with levels of different mediators of inflammation (Li et al. 2021). Overall, methylprednisolone has been established to exhibit positive results in worse conditions of COVID-19 lung inflammation, necessitating a great consideration prior to the episode of ARDS. Even so, prospect randomized controlled tests are urgently needed to verify these outcomes, while additional investigation is required following release (Li et al. 2021).

Corticosteroids, particularly methylprednisolone, have been shown to recover impaired immunity consequent to infection by COVID-19 and augment lower blood pressure (Majmundar et al. 2020). Methylprednisone has been evidenced to provide positive effects towards COVID-19-associated ARDS. In brief, among 50 patients with ARDS treated with methylprednisolone, 23 deaths were recorded, while among 34 others treated differently, 21 deaths were noted (Wang et al. 2020a, b). Moreover, severe cases of COVID-19 that developed to acute respiratory failure were managed successfully using methylprednisolone (Corral et al. 2020). Furthermore, the approved protocol recommended lower or intermediate doses of methylprednisolone and the frequent doses of methylprednisolone usually used varied between 40 and 80 mg administered intravenously during a period of 3 to 6 days (Salton et al. 2020). Nevertheless, the suitable dose, position in treatment, as well as its function necessitated further precision. Unfortunately, it has been documented that the use of corticosteroids may be associated with harmful outcomes towards COVID-19 management (Edalatifard et al. 2020). In a single study, it has not been proved to influence death or diminish microbial elimination (Liu et al. 2020). In addition, it is not suggested in the context of slight pulmonary damage without respiratory syndrome when patients are receiving automatic oxygen therapy (Jeronimo et al. 2021). Notwithstanding the existing slight proof, they advised corticosteroids therapy to manage ARDS. Dexamethasone has been established to decrease oxygen need and death in the context of serious ARDS not associated with COVID-19 (Lammers et al. 2020).

## Mechanism of action in COVID-19

Corticosteroids mainly ciclesonide inhibit virus replication and inflammatory response in the COVID-19 patient through modulation of the viral cytopathic effect, whereas only ciclesonide had an effective viral growth inhibition (Iwabuchi et al. 2020). It has been demonstrated that coronavirus bypass ciclesonide by mutation at A25V in nonstructural protein 15 (NSP15) followed by recombination (Nakajima et al. 2020). Fascinatingly, mometasone was shown to inhibit the mutant virus, hence signifying that its antiviral target was not similar to ciclesonide one (Matsuyama et al. 2020). Then, the probable mechanism of action of ciclesonide was through the quantification of viral RNA (Mori et al. 2021). The effectiveness of ciclesonide and mometasone in the inhibition of viral replication was comparable with the activity of lopinavir (Yamasaki et al. 2020). It was demonstrated that ciclesonide may probably act together with viral NSP15 to suppress viral biogenesis (Al-Kuraishy et al. 2022g). Nevertheless, further investigations were required to fully explain the mechanisms through which ciclesonide suppresses viral biogenesis. It has been demonstrated that ciclesonide is devoid of harmful effects and can be used in children at elevated doses (Tagliati et al. 2021). Ciclesonide principally acts in the lung since it no longer penetrates the circulatory system (Tagliati et al. 2021).

It was demonstrated that ciclesonide exhibited positive outcomes towards COVID-19 (Deokar et al. 2021). Previously ciclesonide was shown to inhibit the MERS virus and inhibited the lung inflammatory process (Terada-Hirashima et al. 2020). Previous reports suggested that the virus penetrated alveolar cells, provoking lung injury and concurrently contaminated macrophages. Therefore, local inflammation could develop, and ciclesonide may presumably play a positive role in such conditions due to the virus causing serious cases (Salvi 2021). To date, no others inhaled steroids have shown antiviral activity against COVID-19, apart from ciclesonide. However, steroid therapy for COVID-19 patients was not suggested given that it may probably prolong viremia and complications like diabetes even though systemic corticoids were recommended (Halpin et al. 2020). In fact, ciclesonide did not reach the circulatory system and remained in the alveolar cells. Thus, should be administered to pneumonia patients prior to the development of severe cases, and was presumed to quickly improve the patient’s symptoms and prevent the development of severe pneumonia (Halpin et al. 2020). The recommended dose for adults ranged between 400 and 800 µg/day. Given that virus, replication time ranged between 6 and 8 h, repeated as well as increased doses were required to obtain an adequate amount of drug in alveoli (Kang and Rhie 2020). Concerning pneumonia in COVID-19 cases, it was reported that three of the six patients who were not administered ciclesonide got worse symptoms and were transported to an intensive care unit, with two (as well as one whose symptoms appeared after hospitalization) under ventilation. Instantaneously after hospitalization, patients with pneumonia developed slightly severe symptoms which gave the impression to deteriorate quickly 7 to 10 days following onset, even though symptoms were preliminary slight. Meanwhile, no respirator was available in the hospital for an infectious disease unit, therefore, exposing the personnel to a serious risk in admitting pneumonia patients with aggravated conditions (Saghazadeh and Rezaei 2020). At the time physicians were looking for a probable destination for a transfer, they were informed that ciclesonide was reported to provide positive outcomes towards SARS-CoV-2, hence began treatment, leading to quick benefit reported in the present study.

**Fig. 3:**
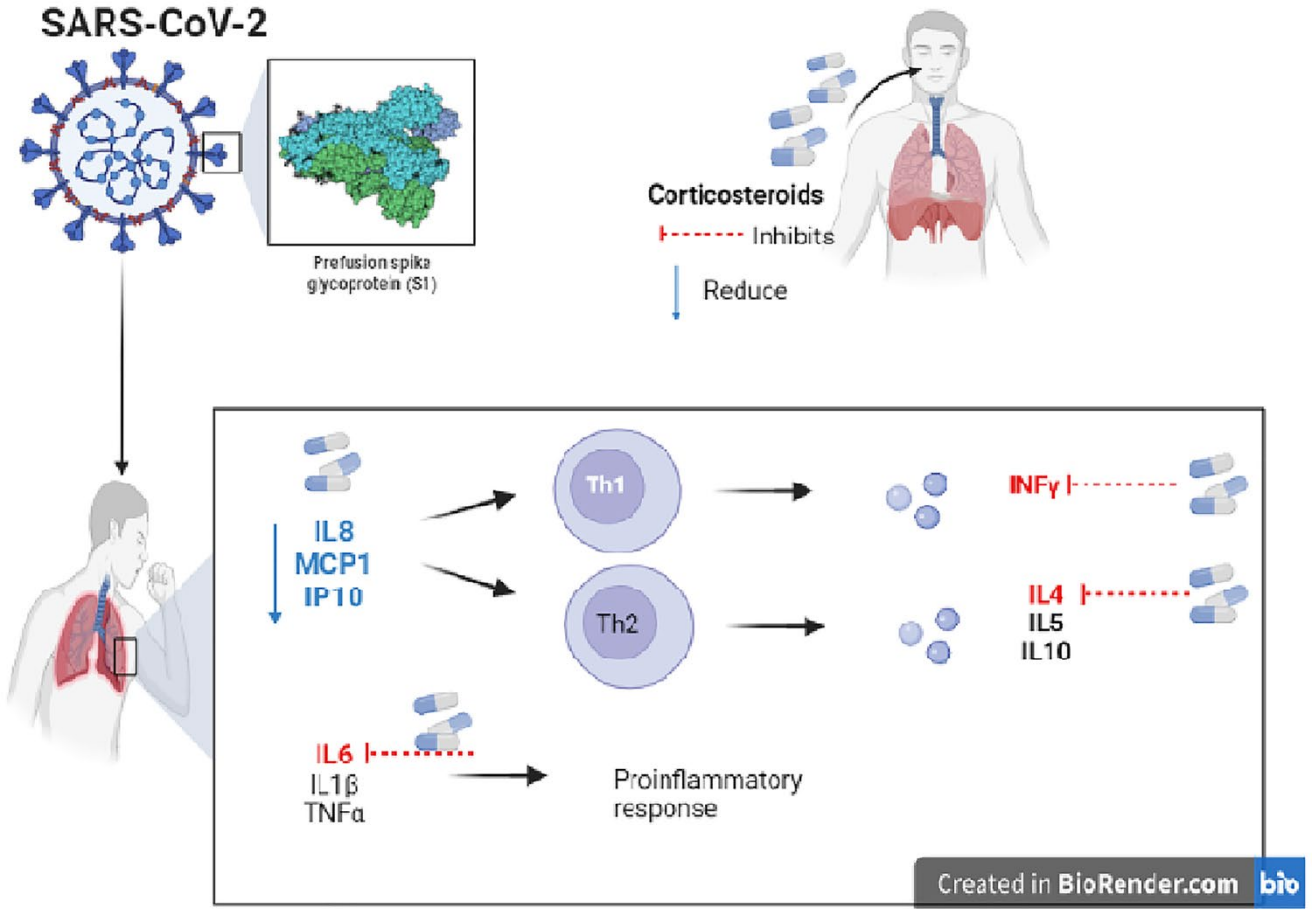
The possible effects of corticosteroids in COVID-19. IL-8 (interleukin 8), MCP-1 (monocyte chemoattractant protein 1), IP-10 (interferon-γ-inducible protein 10), IL-6 (interleukin 6), IFN-γ (interferon-gamma, TNF-α (tumor necrosis factor-alpha) and IL-4 (interleukin 4)

It has been shown that ciclesonide, an inhaled steroid drug, is associated with suppressed asthmatic attacks by reducing airway hyper-activity along with lung resistance following exposure to antigens (Terada-Hirashima et al. 2020). Moreover, it was reported that ciclesonide suppressed the release of inflammatory mediators, slowed down the penetration of immune cells into the lung (Terada-Hirashima et al. 2020). After inhalation, ciclesonide bound to its receptor thereafter exerted effective activities towards inflammation. Moreover, it contained many small size particles that easily reached and infiltrated. Ciclesonide was shown to have low undesirable effects. The peripheral alveoli may be the key site of inflammation as per previous outcomes. Overall, it is here reported that inhaled ciclesonide therapy gave positive results against serious COVID-19 pneumonia (Wong et al. 2021). The net effects of corticosteroids in COVID-19 seem to be beneficial by reducing the release of pro-inflammatory cytokines (Fig. 3).

## Corticosteroids in COVID-19 with asthma

It has been proven that viral lung diseases are among the main frequent causes of exacerbation in asthma and chronic obstructive pulmonary diseases (COPD) patients (Tangirala et al. 2020). Notwithstanding the lack of conclusive results on the potential implication of COVID-19 in exacerbation causes in asthma/COPD, the same stimulation of inflammation and consequent COVID-19’ pneumonia implied a possible exacerbation in asthma/COPD cases (Schultze et al. 2020). The inhaled corticosteroids (ICS) dose in asthma and COVID-19 patients could increase as soon as the situation worsened, along with decreasing the necessity for corticosteroid therapy (Liao et al. 2021). Concerning patients administered combined therapies, another option aimed at increasing dosage as soon as the situation aggravated, that reduced the menace of aggravation necessitating more oral corticosteroids (OCS) than airway administered acute bronchodilator (Liao et al. 2021). It was noted that acute OCS therapy towards asthma and COPD exacerbations quickly improved the situations, avoided illness evolution, untimely declined following urgent care and decreases morbidity. Therefore, OCS treatment could be stopped immediately once signs declined with subsequent amelioration of lung function (Singh and Halpin 2020). Nevertheless, self-initiation of short-term use of OCS should be avoided when common signs of COVID-19 appeared, and procedure previously described in the management of exacerbations has been successfully applied in serious or crucial cases of COVID-19-associated asthma and COPD receiving intensive treatment (Singh and Halpin 2020). Concerning hospitalized patients who did not develop exacerbation so far, but administered long-term treatment of ICS/OCS lasting 3 months consecutive to probable adrenal insufficiency, endocrinologists/diabetologists recommended taking into account a normal systemic corticosteroids dosage that recommended the use of corticosteroids considering the sternness of COVID-19 in sick persons developing both asthma and COPD (Bouazza et al. 2021). Systemic corticosteroids might be beneficial in severe or critical illness, particularly developing septic shock or ARDS, whenever cytokine level increase was confirmed. This was confident with the previous findings, even so, it further needs to be proven. Hence, airway corticosteroids therapy was frequently used, but rarely through nebulizer despite the fact that joint guidance gave advice that nebulized treatment could be used safely (Bouazza et al. 2021). Moreover, the nebulized treatment may be implicated in enhancing contamination in the hospital, hence, metered-dose inhalers with space devices were recommended instead of nebulizers to convey drugs everywhere. Nevertheless, a specialized equipped unit was required for such cases and patients should care with precaution (Rogliani et al. 2020).

ICS therapy was shown to enhance pneumonia risk in patients with COPD, with lipophilic drugs like fluticasone furoate, following its long-term lung stay and related immunosuppressive effect when lung microbiome damage and alteration of mucociliary elimination were established (Edis 2020). In addition, the immunosuppressive effect of fluticasone propionate improved bacterial biofilm consecutive to viral contagion (Edis 2020). Besides, it has been suggested that corticosteroids could decrease the biogenesis of defending molecules in pulmonary cells (Nicolau and Bafadhel 2020). Therefore, further contamination caused increasing lung trouble besides viral pneumonia. Consequently, extrapolating from COPD to asthma should be done with caution, even if in some few cases a reactive effect of eosinophils was noted following corticosteroid administration. For this reason, with an important number of blood immune cells, fluticasone furoate was shown to decrease dyspnea with a noteworthy advantage, when compared with its likely undesirable effect to trigger serious lung inflammation (Çelebioğlu 2020). Even so, ICS therapy was evidenced to enhance lung inflammation when asthma was established. Fascinatingly, preliminary non-peer-reviewed data reported that some corticosteroids suppressed SARS-CoV-2 in vitro with activity comparable with that of lopinavir (Berton et al. 2020). Concerning ciclesonide, the NSP15 has been shown to be its target. Budesonide and formoterol have been shown to autonomously inhibit the systemic suppressive effect of IL-6 causing short-term pulmonary (Pirzada et al. 2021). It was observed that patients with asthma taking ICS were reported to be 49% lesser expected to develop severity consecutive to influenza A/H1/N1 contamination (Halpin et al. 2020) suggesting a common protecting ICS property. Meanwhile, patients with asthma were mainly called to conform to their ICS controller treatment, since this may probably ensure the greatest defense towards viruses like SARS-CoV-2. Unfortunately, no proof was available supporting airway ciclesonide or mometasone in asthmatic persons (Halpin et al. 2020). Further trial about ciclesonide should be investigating the rate of SARS-CoV-2 suppression in moderate COVID-19 while another experiment will focus on the potential effect of elevated dose of ciclesonide versus standard treatment towards serious COVID-19-related lung inflammation. Corticosteroids have been shown to exert a wide-spectrum suppressive effect (Avdeev et al. 2020). Systemic corticosteroids were included in therapy used to control short-term increasing severity in asthma and COPD and were also successful against inflammatory eosinophils (Halpin et al. 2021). However, they revealed an immunosuppressive activity and amplified viral multiplication that additive interferon normally inverted (Robinson and Morand 2021). The upcoming research about interferon-beta-1a will aim at investigating the effectiveness of upregulating pulmonary antiviral defenses against COVID-19 (Kumar et al. 2020). At this time, concurrent experiments will assess interferon-beta-1a given through subcutaneous route with lopinavir/ritonavir. Therefore, it can be hypothesized that nebulized interferon-beta-1a reach sufficient pulmonary drug concentrations towards COVID-19 outbreak like any similar nebulized antibiotics.

## Corticosteroids in COVID-19 with kidney transplantation

About 40 publications have evidenced approaches for the management of hospitalized patients with SARS-CoV-2 in patients receiving renal transplants even if no current-controlled tests are available. For the most part, patients were administered immunosuppressors. Overall, receivers required oxygen. 30 patients received immunosuppressive treatment along with corticosteroids, in general with intravenous methylprednisolone (Johnson et al. 2020). However, a steroid-sparing treatment was administered to the patients; this patient’s immunosuppression was assessed with stopped antiproliferative treatment and decreased tacrolimus; though, methylprednisolone 40 mg/day was as well added during the hospital stay. The completed recovery was observed 2 months following symptoms appearance, 34 of 40 patients received test agents targeting SARS-CoV-2, with 12 diverse approaches trialed among patients (Fadel et al. 2020).

It was reported hereby the effectiveness of steroid immunosuppressive treatment against COVID-19 comprising decreasing dose of antiproliferative treatment along with a slight decline in tacrolimus target in a dose-dependent manner (Chen et al. 2020a, b). Unlike most earlier trials, patients were treated without immunosuppressive corticosteroid before hospitalization, as it was usually done for patients receiving transplants. Based on several reasons, merely slight maintenance immunosuppressive changes were made to find an alternative to corticosteroids in preserving immunosuppression (Chen et al. 2020a, b). Initially, findings after corticosteroids therapy against COVID-19 were merged. Recently, it was recommended that corticosteroids should not be used to control respiratory distress when SARS-CoV-2 is established, only if prescribed for different purposes (Bartiromo et al. 2020). Furthermore, earlier administration of corticosteroids has been linked with a suppressed immune response, a decreased pathogen elimination, and an elevated viral shedding for the period of SARS-CoV-2 outbreak or in the case of moderate illness (Bartiromo et al. 2020). Ultimately, recent outcomes confirmed dissimilar evolution of respiratory disease as well as survival following replacement of elevated doses of corticosteroids used to completely stop calcineurin inhibitor along with antiproliferative treatment (Cheng et al. 2020a, b). Unfortunately, it was reported that corticosteroid therapy used to sustain immunosuppression in these patients increased the risk of infections and deserves carefulness in their use towards transplant SARS-CoV-2 patients (Cheng et al. 2020a, b).

Glucocorticoid regimen was principally monitored considering patient acceptance and biochemical parameters, given that their concentrations are not usually adjusted. Furthermore, the Centers for Disease Control and Prevention (CDC) advised not to use glucocorticoids at an elevated dose in COVID-19 cases since they could probably extend viral replication (Bae et al. 2021). In addition, any current clinical proof evidenced glucocorticoids therapy for pulmonary damage associated with SARS-CoV-2. Nevertheless, patients who commonly used low-dose glucocorticoids for chronic diseases were advised to adopt a conservative but cautious feeling with conservation or a modest decrease of the common dose (Thng et al. 2021).

In a study carried out in Italy, several immunosuppressive patients receiving a kidney transplant were predicted to develop COVID-19. Until now, just a limited number of COVID-19 cases were recorded among them. The patient considered had in general clinical features comparable to those described in non-transplanted cases. While entering ICU, respiratory data were found to be comparable with those of patients not administered immunosuppressive treatment (Chen et al. 2020a, b). However, patients receiving transplants needed more cautious attention considering their impaired immune system with the likely menace of graft loss for the period of SARS-CoV-2 outbreak (Alberici et al. 2020). Therefore, prednisolone dosage was augmented with the withdrawal of other drugs. Considering the effectiveness of steroids in inflammation, their adjustments could be suitable both in decreasing the threat of short-term injection and in alleviating COVID-19 pneumonia, even if its use in such cases was still polemical. Notwithstanding, balancing immunosuppression in COVID-19 was complicated taking into account the necessity of preventing graft loss to avoid too much multiplication of viruses besides reducing immune improvement. Hence, it was recommended to decrease or stop immunosuppressive treatment in kidney transplantation patients for the period of pandemics (Monreal et al. 2021). However, no study has clarified the influence of extensive immunosuppressive therapy during SARS-CoV-2 outbreak, while calcineurin inhibitors (CNIs) could be beneficial in alleviating the stimulation of the natural immunity and stopping the progression of interstitial respiratory illness (Meziyerh et al. 2020). Methylprednisolone administration triggered the failure of the immune system, therefore the withdrawal of immunosuppressive therapy considering a high threat.

The decrease of immunosuppressive therapy and methylprednisolone administered in a reduced dose regimen exhibited positive effects in kidney transplant patients. Steroids along with new therapies were essential for the reestablishment of severely ill patients considering their anti-inflammatory and graft-protective properties (Gandolfini et al. 2020). Initially, a lower dose of calcineurin inhibitor and mild rise in methylprednisolone decreased immunosuppression. However, no current proof has confirmed the significant benefit of corticosteroids given alone in patients with COVID-19 in comparison with corticosteroids associated with another immunosuppressive therapy. No serious signs of COVID-19 were observed even if administering immunosuppressive drugs (Lauterio et al. 2020). The level of pro-inflammatory molecules was noted at day 4 following the withdrawal of immunosuppressive therapy. Corticosteroid therapy may decrease IL-6 levels, currently implicated among key features provoking COVID-19 lung inflammation (Huang et al. 2020). Actually, acute kidney injury (AKI) has been documented as an autonomous forecaster of death consecutive to COVID-19. Nevertheless, the administration of tacrolimus along with ritonavir leads to the rationale effect of glomerular filtration rate following undue administration of tacrolimus. Given that COVID-19 was highly transmissible, it was necessary to manage such patients with caution.

The excessive use of immunosuppressive agents has been documented to noteworthy suppress the T cell immune response of patients receiving renal transplants (Pereira et al. 2020). Consequently, some clinical COVID-19 presentations possibly are typical, and then the management protocol should be done with caution. Concerning the above case, taken as a whole, general presentations, it has been found to be identical to those observed in patients with no symptoms following immunosuppressive therapy (Hasan et al. 2020a, b). On the topic of treatment, the specificity of transplant recipients should be related to the necessity of considering immunosuppressive agents while protecting graft function. The frequent strategy used in the treatment of pneumonia owing to viral contagion after the graft was the diminution or brief discontinuation of immunosuppressants. It gave recipients the occasion to rapidly strengthen their immune system, therefore contributing to viral clearance. Concerning this patient’s case, the treatment initially consisted of discontinuation of immunosuppressive therapy to decrease the brutality of sign evolution related to COVID-19 lung inflammation. After that, in view of the development of the disease and its evolution, immunosuppressive therapy was progressively restored. Nevertheless, before restarting oral immunosuppressants, correct doses of corticosteroids were administered to the patients. Corticosteroids were shown to prevent early graft rejection and avoid crisis following the withdrawal of oral steroids; furthermore, corticosteroids were shown to alleviate inflammation (Domínguez-Gil et al. 2021). Therefore, the dose regimen, as well as the extent of steroid treatment, should be checked. However, overdose and continuous treatment with steroids might not be effective since it has been evidenced to weaken the immune response towards viruses, thereafter might as well lead to other undesirable effects associated with steroids. Therefore, the above outcomes cannot be advised to graft recipients. Further studies are required to improve the procedure of management of immunosuppressed COVID-19 patients.

Overall, immunosuppression might have two divergent effects on COVID-19; first, worsen the acute phase of the illness and delay virus clearance; next, decrease the incidence of deadly serious lung inflammation by suppressing the excessive reactivity of the immune system (Benotmane et al. 2021). The administration of reduced to middle regimen of corticosteroids was suggested in the management of COVID-19 patients with pneumonia, which inhibit the devastating inflammation mediated by the hyperimmune response. Though, concerning steroid regimen and therapies, one should imperatively define a slight set of scales that increases advantages and reduce likely risk (Zhang et al. 2020).

## Corticosteroids in COVID-19 with other comorbidities

### Rheumatoid arthritis

The clinical investigation revealed that systemic corticosteroids effectively reduced immune inflammation. As it is the case in inflammatory rheumatic diseases, corticosteroids can be used as a connection to effective treatment towards COVID-19. Corticosteroid therapy was crucial for rheumatoid arthritis treatment for more than 70 years while it was in recent times improved considering its effectiveness in triggering recovery and connecting therapy to manage illness exacerbation (Gazzaruso et al. 2020). Even though corticosteroids have been shown to quickly suppress inflammatory response throughout the trigger and severe stage of rheumatoid arthritis, their consequence may be the large spectrum of undesirable effects, as well as aggravation of the disease along with the great susceptibility to comorbidities which will increase the exposure to the disease (Guardiola et al. 2020). It was shown that patients with rheumatoid arthritis were highly exposed to all the diseases after corticosteroid therapy at varying doses, though rheumatoid arthritis patients administered corticosteroids were highly exposed to microbial contaminations. To illustrate this, it was shown that patients treated with corticosteroids are highly exposed to Herpes Zoster diseases (Roongta et al. 2020). Hence, corticosteroids have been evidenced to hinder host defense as well as retard microbial elimination, whereas they also contained inflammation that principally caused lung injury and ARDS outbreak (Song et al. 2020). In fact, a recent provisional regulation on the management of COVID-19 does not recommend the use of corticosteroids except otherwise specified.

## Inflammatory bowel diseases (IBDs)

Corticosteroid therapy, immunomodulatory drugs, as well as monoclonal antibodies were generally used to trigger the improvement in IBDs, known as immune-mediated illnesses. Unfortunately, the above drugs were shown to likely exacerbate the susceptibility to secondary diseases principally (Brenner et al. 2020). For that reason, one should adopt convenient decisions in avoiding and caring for sick persons, which constitute a key stool in the protocol of management of IBD. A difficult decision has been taken to suppress infliximab and immunosuppressant use in the IBD context (Mazza et al. 2020). In fact, a systematic review reported a considerably diminishing of hospitalization following IBD therapy. The possibility of deterioration following the suppression of successful administration of the above therapies was around 50% was linked to an augmented requirement for steroids, and likely hospitalization and surgical intervention (Iacucci et al. 2020). However, several aspects should be considered. First, SARS-CoV-2 is a serious disease but not an opportunistic one, given that no susceptibility to the disease was associated with simultaneous immune deficiency (Cappello et al. 2020). Second, serious COVID-19 was shown to be associated with cytokine storm and likely related to the hyper-activity of host defense besides a microbial-associated injury. Thirdly, approximately 5% of cases that worsen following the withdrawal of efficient treatments will need hospitalization against the scenery of overcame hospital capability. Therefore, the disadvantage and advantage of pursuing or suppressing biological therapy require cautious analysis and are different from the usual regulation used in IBD, particularly specifying the expected duration of the pandemic (Lukin et al. 2020).

Recently published data had confirmed that several immunotherapies in IBD patients induced and augmented susceptibility to the diseases since they have been reported to suppress host defense (Turner et al. 2020). Corticosteroids have been coupled with high susceptibility to the diseases and were largely experienced in managing ARDS and excessive inflammatory response consecutive to COVID-19. Even though these reports did not aim at investigating steroid efficacy, it was found that methylprednisolone therapy diminishes mortality owing to COVID-19 (Burke et al. 2021).

A recent study has investigated the features of COVID-19 in subjects with IBD along with the link between populations, symptoms, and immunosuppressive therapy in COVID-19 was assessed. Hence, it was reported that increasing age, comorbidities, systemic corticosteroids, and sulfasalazine or 5-aminosalicylate use worsen the situation of COVID-19 cases (Mak et al. 2020). Moreover, it is necessary to maintain decreasing steroid doses in controlling IBD during the outbreak.

## Conclusion

Corticosteroid therapy is an effective way in the management of COVID-19; it reduces the risk of complications mainly acute lung injury and the development of ARDS. Besides, corticosteroid therapy mainly dexamethasone and methylprednisolone are effective in reducing the severity of COVID-19 and associated comorbidities such as COPD, rheumatoid arthritis and IBD.

## Data Availability

Not applicable.
